# Mandibular nerve schwannoma: a case report

**DOI:** 10.11604/pamj.2022.42.24.34713

**Published:** 2022-05-11

**Authors:** Jinane Kharbouch, Zakaria Aziz, Mohamed Lahrach, Yassine Bennaoui, Salma Aboulouidad, Mohamed El Bouihi, Nadia Mansouri Hattab

**Affiliations:** 1 Maxillo-Facial Surgery Department, University Hospital Center Mohammed VI, Marrakech, Morocco

**Keywords:** Schwannoma, intraosseous, mandible, inferior alveolar nerve, case report

## Abstract

Benign schwannomas are uncommon and their intraosseous location is even rarer counting for less than 1% of all benign primary bone tumors. They exceptionally occur in the oral cavity with the tongue being the most common site of involvement. We report here a case of intramandibular schwannoma derived from the inferior alveolar nerve, in a 57-year-old patient with a 3 months history of inferior left lip paresthesia. The oral examination showed a firm, painless and non-pulsatile swelling located in the inferior vestibule. The panoramic X-ray revealed a circumscribed and homogeneous radiolucent image. Treatment consisted of total excision of the tumor with preservation of the nerve bundles. The histological examination confirmed the diagnosis of schwannoma. The patient recovered a normal sensory function 6 months post-operatively without any recurrence up to 2 years after surgery. The treatment of intramandibular schwannoma is basically surgical with the conservative approach being the most advocated by majority of authors.

## Introduction

Schwannomas, also called neuromas or neurilemomas, are benign nerve tumors developed from schwann cells, which forms the myelin sheath surrounding the axons of peripheral nerves. This myelin sheath allows a faster and saltatory conduction velocity in the axons involved [1,2].

Schwannomas are uncommon and their intraosseous location is even rarer counting for less than 1% of all benign primary bone tumors [3]. Mandibular intraosseous schwannomas are developed from alveolar nerve. Due to their slow progression, they are most often asymptomatic and discovered only after a fairly significant growth or following the gradual developing of a sensory disorder [1].

We report here a unique case of a mandibular intraosseous schwannoma and its surgical management.

## Patient and observation

**Patient information:** a 57-years-old patient, with no particular pathological history, referred to us for paresthesias of the inferior alveolar nerve evolving since 3 months.

**Clinical findings:** exobuccal clinical examination did not find any swelling or lymphadenopathy, and Vincent's sign was positive. The intrabuccal examination showed a small painless vestibular lump on the left mandible, covered with a healthy-looking mucosa. The patient was totally toothless.

**Diagnostic assessment:** the orthopantomogram revealed a circumscribed, homogeneous radiolucent image, well limited by a clear and continuous condensing wall, on the left horizontal branch. The mandibular canal appears to be enlarged in contact with the lesion.

**Figure 1:**
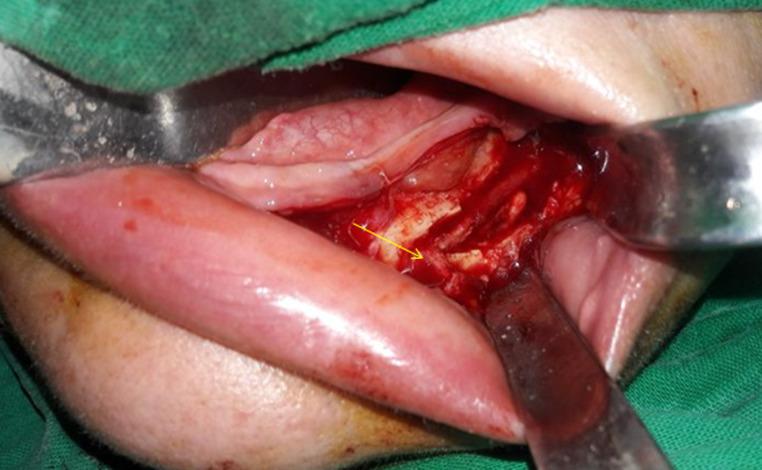
peroperative view showing the residual after surgical excision of the tumor with preservation of alveolar nerve (arrow)

**Therapeutic intervention:** excision of the lesion was performed under general anesthesia after surgical breach of the external mandibular cortical bone, revealing a lesion surrounded by a fibrous capsule in the mandibular canal. Although the lesion was connected to the lower alveolar neurovascular bundle, it easily detached from the nerve fibers, allowing them to be preserved (Figure 1).

The mass and the fragments were sent for pathological examination, which confirmed the diagnosis of a schwannoma as it displayed areas of cell proliferation of moderate density interspersed with predominantly lymphocyte tissue within a fibrohyaline shell. The Antoni A areas were predominant; made of spindle-shaped and bipolar, epithelioid, well-organized cells grouped in parallel, arranged in clusters, with a compact palisade-like arrangement of nuclei within a collagen-rich connective tissue; occasionally alternating with the Antoni B areas.

**Follow-up:** postoperatively, the patient maintained left labiomental paresthesia for a period of 6 months before its total resolution, supported by vitamin supplementation. Up to two years post-operatively, no recurrence was noted.

## Discussion

The schwannoma´s most common location is the upper and lower limbs, they exceptionally occur in the oral cavity with the tongue being the most common site of involvement [4]. Intraosseous schwannomas are rare, the mandible is the site most often affected, corresponding to the intraosseous trajectory of the lower alveolar nerve [5].

They occur at any age, most often in the fourth and fifth decades with a female predilection. They are generally asymptomatic and have a slow progression. Exceptionally, they can cause aesthetic or functional repercussions. The latter can range from a simple transient primary local paresthesia, same as in our patient, to a total loss of sensitivity of the inferior alveolar nerve [5]. Radiologically, these tumors present as a radiolucent, unilocular, homogenous and well limited image by a bony wall with cortical expansion. These features are common to other lesions such as odontogenic cysts, intraosseous angioma, eosinophilic granuloma, or even ameloblastoma [6]. Only pathological examination can confirm the diagnosis of intraosseous schwannoma.

Histologically, the tumor is surrounded by a fibrous capsule consisting of two cell populations; the first so-called Antoni A type cell population which is characterized by a hypercellular proliferation of bipolar fusiform elements, within a rich network of collagen fibers and reticulin. The cells group together in such a way that the nuclei form a palisade. The juxtaposition of several palisades, like a barrel stave, is known as the verocay nodule. The second population, known as the Antoni B type, concerns fewer and less well-ordered cells within a looser connective tissue. The cells are more rounded with reduced cytoplasm and rounded nucleus resembling lymphocytes. This shape could represent type A degeneration [7].

Conservative surgery is the treatment of choice, consisting of excision or enucleation of the tumor. Schwannomas are well encapsulated, ovoid, eccentric with respect to the nerve trunk and their excision is carried out without any difficulty. Nerve preservation throughout surgical removal deserves special attention from the surgeon. The literature suggests that the nerve should be separated from these tumors and preserved when the lesion is removed [8]. However, a few reports indicate that the nerve may be resected due to the possible risk of recurrence even if the tumor is benign [9]. In general, the risk of recurrence and malignant transformation remains very low to exceptional [10]. In our case, careful dissection and stripping of nerve bundles was performed to preserve nerve function. Radiotherapy has no place in the therapeutic management because these tumors are radioresistant [4].

## Conclusion

Reported cases of mandibular schwannoma remain very rare. This benign neurogenic tumor requires a thorough anamnesis, clinical examination, imaging and pathological examination to obtain the histological origin. Its treatment is based on surgical resection with a conservative approach to the nerve bundles whenever possible.
